# Omentin-1 Val109Asp polymorphism and increased coronary artery disease risk in smokers with type 2 diabetes

**DOI:** 10.1097/MD.0000000000049405

**Published:** 2026-06-19

**Authors:** Dahong Yu, Linchao Tong, Xiaowei Ma, Nan Gu, Difei Lu, Na Yu, Yuxin Wang, Junqing Zhang, Jianping Li, Xiaohui Guo

**Affiliations:** a Department of Endocrinology, Peking University First Hospital, Beijing, China; b Department of Endocrinology, Changping District Hospital, Beijing, China; c Department of Cardiology, Peking University First Hospital, Beijing, China.

**Keywords:** coronary artery disease, gene–environment interaction, omentin-1, single-nucleotide polymorphism, type 2 diabetes mellitus

## Abstract

Omentin-1 protein has protective effects against coronary artery disease (CAD). The Val109Asp polymorphism (rs2274907) in the Omentin-1 gene has been associated with CAD risk, but this relationship has not been investigated in patients with type 2 diabetes mellitus (T2DM). To investigate the association between the Omentin-1 Val109Asp polymorphism and CAD risk in Han Chinese patients with T2DM. This case-control study enrolled 465 Han Chinese patients with T2DM from July 2008 to September 2015. Participants were classified as CAD-positive (coronary stenosis > 50% in at least 1 major vessel, n = 249) or CAD-negative (n = 216). The Val109Asp polymorphism was genotyped using PCR-RFLP. Logistic regression analysis was performed to assess the association between genotypes and CAD risk, with adjustment for potential confounders including age, sex, smoking status, hypertension, dyslipidemia, diabetes duration, body mass index, and fasting glucose. The A allele frequency was higher in CAD patients than in controls (34.5% vs 27.5%; OR = 1.386; *P* = .026). Under the dominant model, TA/AA carriers had increased CAD risk compared with TT homozygotes (adjusted OR = 1.509, 95% CI: 1.025–2.222; *P* = .037). Stratified analyses showed that smokers carrying the A allele exhibited further elevated CAD risk (adjusted OR = 1.82, 95% CI: 1.02–3.27). The Omentin-1 Val109Asp polymorphism is associated with increased CAD risk in Han Chinese patients with T2DM under the dominant genetic model. Stratified findings suggest that smoking may further heighten CAD susceptibility among A-allele carriers. Integrating genetic and lifestyle factors may improve cardiovascular risk assessment in this population.

## 1. Introduction

Type 2 diabetes mellitus (T2DM) affects approximately 537 million adults worldwide and is projected to reach 783 million by 2045.^[1]^ This metabolic disorder is characterized by insulin resistance and progressive β-cell dysfunction, leading to chronic hyperglycemia and increased risk of micro- and macrovascular complications.^[2]^ Coronary artery disease (CAD) represents the leading cause of morbidity and mortality among patients with T2DM, with cardiovascular mortality rates markedly higher than in nondiabetic individuals.^[3]^ While traditional risk factors such as hypertension, dyslipidemia, and smoking contribute to CAD development, substantial inter-individual variation in cardiovascular outcomes among T2DM patients suggests an important role for underlying genetic susceptibility.^[4]^ Moreover, T2DM patients frequently exhibit metabolic syndrome – a cluster of insulin resistance, central obesity, dyslipidemia, hypertension, and triglyceride-glucose index abnormalities – which amplifies cardiovascular risk.^[5,6]^

Omentin-1 is a 313-amino acid adipokine predominantly secreted by visceral adipose tissue, particularly epicardial adipose tissue surrounding coronary arteries.^[7]^ The omentin-1 gene is located on chromosome 1q23.3 and encodes a protein with anti-inflammatory, vasculoprotective, and insulin-sensitizingproperties.^[8]^ Multiple studies have demonstrated that circulating omentin-1 levels are significantly reduced in patients with CAD compared to healthy controls and correlate inversely with coronary atherosclerotic burden.^[9,10]^ Similarly, serum omentin-1 concentrations are decreased in T2DM patients, suggesting a shared pathophysiological link between reduced omentin-1 activity and both metabolic and cardiovascular dysfunction.^[11]^

The missense single-nucleotide polymorphism Val109Asp (rs2274907) in the omentin-1 gene results in substitution of valine (hydrophobic amino acid) with aspartic acid (negatively charged amino acid) at position 109, which may alter protein structure and function.^[12]^ Several studies have investigated the association between this polymorphism and CAD risk, but findings have been inconsistent across different populations. In Iranian and Iraqi populations, the A allele (Asp109) was associated with increased CAD risk.^[13,14]^ Conversely, studies in Pakistani and Turkish populations reported that carriers of the T allele (Val109) had higher CAD susceptibility.^[15,16]^ These discrepancies may reflect population-specific genetic backgrounds, linkage disequilibrium patterns, or gene–environment interactions that have not been adequately explored.

Importantly, although prior studies have explored the Val109Asp polymorphism in various populations, few have specifically assessed its association with CAD risk among patients with T2DM, which group characterized by heightened metabolic inflammation and elevated baseline cardiovascular risk that may modify genetic effects. Furthermore, gene–environment interactions involving this polymorphism, particularly with major modifiable risk factors such as smoking, have rarely been evaluated in previous research. Given the high prevalence of both T2DM and CAD in the Chinese population and the established role of omentin-1 in cardiometabolic regulation, we conducted this study to investigate the association between the omentin-1 Val109Asp polymorphism and CAD risk in Han Chinese patients with T2DM, with comprehensive evaluation of all major Mendelian inheritance models and specific attention to potential gene–environment interactions.

## 2. Material and methods

### 2.1. Study design and participants

This case-control study was conducted at Peking University First Hospital between July 2008 and September 2015. The study protocol was approved by the Institutional Review Board of Peking University First Hospital (approval number: [2008]092) and conducted in accordance with the Declaration of Helsinki. Written informed consent was obtained from all participants prior to enrollment.

#### 2.1.1. Inclusion criteria

Han Chinese ethnicity; T2DM diagnosis according to the 1999 World Health Organization criteria; and availability of coronary angiography or multi-detector computed tomography coronary angiography results.

#### 2.1.2. Exclusion criteria

Type 1 diabetes mellitus or other specific types of diabetes; active inflammatory conditions, chronic inflammatory disorders, or autoimmune diseases; history of malignancy, immunosuppressive drug use, or known hematological disorders; and previous myocardial infarction or coronary revascularization procedures.

### 2.2. Case and control definition

Participants were classified into 2 groups based on coronary angiographic findings. CAD-positive cases were defined as having ≥50% luminal stenosis in at least 1 major epicardial coronary artery or its main branches. CAD-negative controls were defined as having <50% stenosis in all major coronary arteries and main branches. All coronary angiographic interpretations were performed by experienced cardiologists blinded to genetic information.

### 2.3. Clinical data collection

Demographic and clinical data were extracted from medical records, including age, sex, body mass index, T2DM duration, smoking status, and comorbidities. Hypertension was defined as blood pressure ≥ 140/90 mm Hg or current use of antihypertensive medications. Dyslipidemia was defined according to standard clinical criteria or current use of lipid-lowering therapy. Smoking status was categorized as current smoker or nonsmoker (including never smokers and former smokers who quit >1 year prior to enrollment). Laboratory parameters included fasting plasma glucose and glycated hemoglobin.

### 2.4. DNA extraction and genotyping

Genomic DNA was extracted from peripheral blood leukocytes using the standard salting-out method and stored at −20°C until analysis. The Val109Asp polymorphism (rs2274907, *T* > A) located in exon 4 of the omentin-1 gene was genotyped using polymerase chain reaction-restriction fragment length polymorphism (PCR-RFLP).

#### 2.4.1. PCR amplification

The forward primer sequence was 5′-GAG CCT TTA GGC CAT GTC TCT-3′ and the reverse primer was 5′-CTC TCT TCT TCT CCA GCC CAT-3′. PCR was performed in a 25 μL reaction volume containing 1 μL DNA template, 1 μL each primer (5 pmol/μL), 12.5 μL of 2 × PCR Mix containing Taq polymerase and dNTPs (Beijing Tianyihuiyuan Life Science and Technology Inc.), and 10.5 μL double-distilled water. The PCR cycling conditions were: initial denaturation at 94°C for 5 minutes, followed by 35 cycles of denaturation at 94°C for 30 seconds, annealing at 62°C for 30 seconds, and extension at 72°C for 30 seconds, with a final extension at 72°C for 5 minutes.

#### 2.4.2. RFLP analysis

The PCR products were digested using FokI restriction enzyme (New England Biolabs) in a 20 μL reaction mixture containing 7.5 μL PCR product, 0.5 μL FokI enzyme, 2 μL 10 × reaction buffer, and 10 μL double-distilled water. The reaction was incubated at 60°C for 2 hours, and products were analyzed by agarose gel electrophoresis.

#### 2.4.3. Quality control

Direct DNA sequencing was performed on 5% of randomly selected samples to validate genotyping accuracy. Laboratory personnel were blinded to case-control status during genotyping procedures.

### 2.5. Statistical analysis

Statistical analyses were performed using R 4.5.1 (R Foundation for Statistical Computing, Vienna, Austria). Continuous variables are presented as mean ± standard deviation and were compared using Student *t* test for normally distributed data or the Mann–Whitney *U* test for non-normally distributed data. Categorical variables were summarized as frequencies (percentages) and compared using the chi-square test.

Hardy–Weinberg equilibrium (HWE) was assessed using the chi-square goodness-of-fit test. The association between the Val109Asp genotypes and CAD risk was evaluated using logistic regression analysis under 5 major Mendelian inheritance models: allelic, dominant, recessive, codominant, and heterozygote advantage models.

Multivariate logistic regression was performed with adjustment for age, sex, smoking status, and other clinical covariates as appropriate. Gene–environment interactions were evaluated by including genotype × smoking interaction terms in the regression models. Receiver operating characteristic (ROC) curves were generated to assess the incremental discriminative value of adding genotype to the clinical model. All statistical tests were 2-sided, and a *P*-value < .05 was considered statistically significant.

## 3. Results

### 3.1. Study population characteristics

A total of 465 Han Chinese patients with T2DM were enrolled, including 249 CAD-positive and 216 CAD-negative individuals. The baseline characteristics of the participants are summarized in Table 1. Compared with the CAD-negative group, patients with CAD were significantly older (65.26 ± 10.02 vs 61.37 ± 11.31 years, *P* = .001), had a higher proportion of males (62.3% vs 44.4%, *P* < .001), and were more likely to be current smokers (49.6% vs 40.0%, *P* = .001). No significant differences were observed between the 2 groups in T2DM duration, hypertension, body mass index, glycated hemoglobin, fasting plasma glucose, or dyslipidemia (all *P* > .05), as shown in Table 1.

**Table 1:** Clinical characteristics of CAD cases and controls.

Characteristic	CAD (n = 249)	Non-CAD (n = 216)	*P* value
Gender (M/F)	155/94*	96/120	<.001
Age (yr)	65.26 ± 10.02	61.37 ± 11.31	.001
DM duration (yr)	8.32 ± 7.30	7.13 ± 5.85	.769
Hypertension (%)	77.8	71.2	.11
BMI (kg/m^2^)	25.76 ± 3.27	25.70 ± 2.82	.472
HbA1c (%)	7.20 ± 1.20	7.11 ± 1.15	.08
FPG (mmol/L)	7.30 ± 2.64	6.88 ± 1.70	.263
Dyslipidemia (%)	87.5 (n = 218)	84.7 (n = 183)	.615
Smoking (%)	49.6* (n = 124)	40 (n = 86)	.001

Data are presented as mean ± SD, median (interquartile range), or n (%).

BMI = body mass index, CAD = coronary artery disease, DM = diabetes mellitus, FPG = fasting plasma glucose, HbA1c = Glycated hemoglobin, SD = standard deviation.

* *P* values were obtained by independent samples *t* test, Mann–Whitney *U* test, or chi-squared test.

### 3.2. *Genotype distribution and Hardy–Weinberg equilibrium*

The genotype distribution of the Val109Asp polymorphism was TT (n = 193), TA (n = 245), and AA (n = 27). HWE analysis showed that the CAD-negative control group conformed to equilibrium (*P* = .072), indicating adequate population representativeness and genotyping reliability. In contrast, both the overall sample (*P* = 6.76 × 10^−6^) and the CAD-positive group (*P* = 1.20 × 10^−5^) deviated from HWE, which is expected in disease-enriched cases and does not indicate methodological error. Because the control group met HWE, all genotype categories (TT, TA, AA) were retained in subsequent analyses as required for accurate evaluation of all Mendelian inheritance models. These results are presented in Table 2.

**Table 2:** Hardy–Weinberg equilibrium test for Val109Asp genotype distribution in overall, CAD, and non-CAD populations.

Group	TT	TA	AA	HWE *P*-value
Overall	193	245	27	6.76 × 10^−6^ ****
CAD+	92	143	14	1.20 × 10^−5^ ****
CAD-	101	102	13	0.071

CAD = coronary artery disease, HWE = Hardy–Weinberg equilibrium.

****P < .0001.

#### 3.2.1. Genotype and allele frequency differences between CAD and non-CAD groups

Genotype frequencies significantly differed between CAD and non-CAD groups (TT, TA, and AA: 36.7%, 57.6%, 5.7% in CAD vs 49.5%, 46.0%, 4.5% in controls; χ^2^ = 7.24, *P* = .027), as shown in Table 2.

The A allele frequency was also higher in CAD patients (34.5%) than in controls (27.5%), with an unadjusted odds ratio (OR) of 1.386 (95% CI: 1.039–1.850, *P* = .026).

### 3.3. Genetic association analyses under multiple Mendelian inheritance models

Results from all Mendelian inheritance models are summarized in Table 3.

**Table 3:** Logistic regression results under different genetic models for the Val109Asp polymorphism (adjusted for age, gender, and smoking).

Model	OR	LCL	UCL	*P*
Dominant (TA + AA vs TT)	1.509	1.025	2.222	.0371*
Recessive (AA vs TT + TA)	0.950	0.423	2.133	.9010
Overdominant (TA vs AA + TT)	1.512	1.031	2.216	.0342*
Codominant (TA vs TT)	1.547	1.041	2.299	.0308**
Codominant (AA vs TT)	1.209	0.523	2.795	.6570

LCL = lower confidence limit, OR = odds ratio, UCL = upper confidence limit.

*P < .05.

**P < .01.

#### 3.3.1. Allelic model.

In the allelic model (A vs T), the A allele showed a modest elevation of CAD risk, although the association did not remain significant after covariate adjustment.

#### 3.3.2. Dominant model (TA + AA vs TT).

In the dominant model, A-allele carriers had significantly increased CAD risk (OR = 1.509, 95% CI: 1.025–2.222, *P* = .037), as shown in Table 3.

#### 3.3.3. Recessive model (AA vs TT + TA).

The recessive model showed no significant association (adjusted OR = 0.950, *P* = .901).

#### 3.3.4. Codominant model.

In the codominant model, only the TA genotype showed significance (TA vs TT: OR = 1.547, 95% CI: 1.041–2.299, *P* = .031), whereas the AA genotype did not differ significantly from TT (OR = 1.209, 95% CI: 0.523–2.795, *P* = .657).

#### 3.3.5. Heterozygote advantage.

The heterozygote advantage model (TA vs TT + AA) also showed increased CAD risk (OR = 1.512, 95% CI: 1.031–2.216, *P* = .034), indicating elevated risk among heterozygous individuals.

### 3.4. Gene-smoking interaction and stratified analyses

The interaction term between Val109Asp genotype and smoking status was not statistically significant in multivariable logistic regression (interaction *P* = .514). However, stratified analyses revealed important patterns.

**Figure 1.**
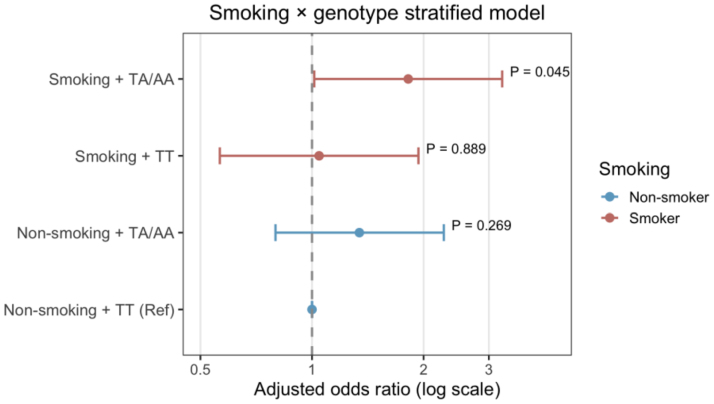
Stratified effects of Val109Asp genotype and smoking on CAD risk. ORs adjusted for age and gender; reference group = nonsmokers with TT genotype. OR = odds ratio.

Among nonsmokers, TA/AA carriers showed a nonsignificant trend toward higher CAD risk compared with nonsmoking TT carriers (adjusted OR = 1.34, 95% CI: 0.80–2.27). Among smokers with the TT genotype, CAD risk did not significantly differ from the reference group (adjusted OR = 1.04, 95% CI: 0.56–1.94). Notably, smokers carrying the A allele (TA/AA) exhibited a significantly elevated CAD risk (adjusted OR = 1.82, 95% CI: 1.02–3.27), suggesting a cumulative effect of smoking and A-allele carriage, although this pattern did not reach significance in the formal interaction test. The stratified associations between smoking, Val109Asp genotype, and CAD risk are detailed in Table 4 and depicted in Figure 1.

**Table 4:** Stratified analysis of CAD risk by Val109Asp genotype and smoking status (adjusted for age and gender).

Group	Adjusted OR (95% CI)	*P* value
Nonsmoker + TT	Reference	–
Nonsmoker + TA/AA	1.34 (0.80–2.27)	.269
Smoker + TT	1.04 (0.56–1.94)	.889
Smoker + TA/AA	1.82 (1.02–3.27)	.045

CAD = coronary artery disease.

### 3.5. Predictive value of Val109Asp genotype

To evaluate the predictive value of the Val109Asp genotype, ROC analyses were performed. As shown in Table 5, the clinical model including age, sex, and smoking yielded an AUC of 0.663. Incorporation of the Val109Asp genotype increased the AUC to 0.672; however, the improvement was not statistically significant (DeLong test, *P = *.26), indicating that although the polymorphism contributes to disease susceptibility, it adds limited discrimination to clinical risk prediction.

**Table 5:** ROC analysis for clinical model and genotype-added model.

Model	AUC
Clinical model	0.663
Clinical + genotype	0.672
DeLong test *P*	0.26

ROC = receiver operating characteristic.

## 4. Discussion

This study investigated the association between the omentin-1 Val109Asp polymorphism and CAD risk in Han Chinese patients with T2DM. Our findings demonstrate that carriers of variant alleles (T/A + A/A genotypes) have significantly increased CAD risk under the dominant genetic model, with the association remaining significant after adjustment for traditional cardiovascular risk factors. Although the formal gene-smoking interaction term was not statistically significant (*P* = .514), stratified analyses showed that smokers carrying the A allele exhibited higher CAD risk (OR = 1.82, 95% CI: 1.02–3.27), suggesting that environmental factors such as smoking may amplify underlying genetic susceptibility.

### 4.1. Comparison with previous studies

The association between omentin-1 Val109Asp polymorphism and CAD risk has been investigated in several populations with conflicting results. Our findings align with studies in Iranian and Iraqi populations, where the A allele (Asp109) was associated with increased CAD risk.^[13,14]^ Conversely, studies in Pakistani, Indian, and Turkish populations reported that the T allele (Val109) conferred higher CAD susceptibility.^[15-17]^ These discrepancies may reflect population-specific genetic architecture, differences in linkage disequilibrium patterns, or varying environmental exposures across study populations.

The Val109Asp substitution involves the replacement of a hydrophobic amino acid (valine) with a negatively charged residue (aspartic acid), which could potentially alter protein conformation and function. Although this biochemical substitution suggests possible structural or functional consequences for omentin-1, direct functional evidence remains limited, and no experimental studies have confirmed the impact of the Val109Asp variant on protein activity or secretion.

### 4.2. *Gene–environment interaction*

The stratified findings suggest that smoking may amplify the genetic susceptibility conferred by the A allele, with smokers carrying the TA/AA genotypes exhibiting higher CAD risk compared with nonsmoking TT carriers, indicating a potential cumulative effect of genetic and environmental factors. Several mechanisms may underlie this pattern. Smoking induces oxidative stress, endothelial dysfunction, and systemic inflammation, which could exacerbate any adverse vascular effects associated with altered omentin-1 function. Moreover, tobacco exposure may influence omentin-1 expression or activity through epigenetic or transcriptional pathways, thereby intensifying the functional consequences of the Val109Asp variant.^[18]^ Second, tobacco exposure may induce epigenetic modifications that regulate omentin-1 transcription, potentially amplifying the downstream functional consequences of the Val109Asp substitution.^[19]^

The pattern observed in our stratified analysis underscores the importance of accounting for environmental factors when interpreting genetic associations with complex diseases. Differences in smoking prevalence and exposure intensity across populations may partly explain the inconsistent findings reported in previous studies.^[20]^

### 4.3. Clinical implications

Our findings suggest that genetic information may offer additional value for cardiovascular risk assessment in T2DM patients, especially when considered alongside smoking status. Smokers carrying the A allele may constitute a particularly vulnerable subgroup with elevated CAD risk and thus may benefit from intensified smoking cessation support and closer cardiovascular monitoring. These observations highlight the potential utility of integrating genetic and environmental risk factors in personalized prevention strategies. However, the broader clinical application of Val109Asp genotyping will require validation in larger, independent cohorts and evaluation of its cost-effectiveness in routine practice.

### 4.4. Study limitations

Several limitations should be acknowledged. First, our sample size was relatively modest, which may have limited statistical power for detecting smaller genetic effects and gene–environment interactions. Second, the cross-sectional design precludes determination of causality and temporal relationships. Third, we focused on a single genetic variant in 1 candidate gene, whereas CAD is a polygenic trait influenced by multiple genetic loci. Fourth, we did not measure circulating omentin-1 levels, which would have provided insight into the functional consequences of the genetic variant. Finally, our findings are limited to Han Chinese patients with T2DM and may not be generalizable to other populations or nondiabetic individuals.

### 4.5. Future directions

Larger prospective studies in diverse populations are needed to validate our findings and establish the clinical utility of omentin-1 genetic testing. Functional studies examining the biological effects of the Val109Asp variant on protein structure, expression, and activity would provide mechanistic insights. Additionally, investigation of multiple omentin-1 variants and their interactions with various environmental factors could enhance understanding of this gene’s role in cardiovascular disease susceptibility.^[21]^

## 5. Conclusion

The omentin-1 Val109Asp polymorphism is associated with increased CAD risk in Han Chinese patients with T2DM under the dominant genetic model. Stratified analyses further suggest that smokers carrying the A allele may represent a subgroup with particularly elevated susceptibility. These findings enhance understanding of the genetic contribution to CAD in diabetes and highlight the value of integrating genetic and environmental factors in cardiovascular risk evaluation.

## Author contributions

**Conceptualization:** Dahong Yu, Xiaowei Ma.

**Formal analysis:** Dahong Yu.

**Investigation:** Dahong Yu.

**Methodology:** Dahong Yu, Linchao Tong, Xiaowei Ma.

**Project administration:** Dahong Yu, Xiaowei Ma.

**Validation:** Dahong Yu, Nan Gu, Difei Lu, Na Yu, Yuxin Wang, Junqing Zhang, Jianping Li, Xiaohui Guo.

**Visualization:** Dahong Yu.

**Writing – original draft:** Dahong Yu.

**Writing – review & editing:** Dahong Yu, Linchao Tong, Xiaowei Ma.
